# Ex-Soldiers and Trade Unions

**Published:** 1920-02-28

**Authors:** 


THE PUBLIC WELL-BEING—*(continued)*

### Ex-Soldiers and Trade Unions.

### Demand for Fair Play.

Rarely has Labour made so sorry a show as it did in last week's Parliamentary debate on the action of certain unions in restricting the employment of discharged and disabled soldiers. On every ground of humanity, sound policy, expediency, and in gratitude for men who have gone through hell for the country's defence, it is mean and unwise in the national and social interest that the doors of industry should be closed against them. Is the man who was fortunate enough to remain at home and earn high wages entitled to behave as though he were an eternally privileged being without consideration for the less fortunate man who went through blood and terror at the front? Those who tended and nursed the soldiers and who know what they went through and with what cheerful courage they faced hardship and suffering, will entertain no sympathy for the selfishness of industrial discrimination against them.

Labour speaks in grandiose terms of the human rights and liberties. It issues moving manifestoes for starving children in Europe. Its political programme is full of ideals and virtuous aspirations. But an ounce of practice is better than a universe of profession. Let Labour start at home to give a chance—just to give a chance, and nothing more—to the men who fought, and to their families. It must have taken a stiff skin to resist the moving appeal made by Major Cohen, a legless ex-Service member of the House, on behalf of the men who had suffered. Cases given by Captain Hambro included one in which a strike occurred in a certain trade because of the employment of an officer, two non-commissioned officers, and a private. An ex-soldier was offered a good post in a shipbuilding yard, provided he joined the trade union, but his application for membership was refused. In another case an ex-soldier had to leave his employment because his retention would cause a strike. Major Hamilton gave the instance of the electrical trade union which refused to admit trained electrical naval ratings, of a branch of the Ironmoulders' Union at Leeds who refused admission to discharged or disabled soldiers unless under sixteen years of age, and he said he could quote instance after instance where strikes had occurred because employers had brought in ex-soldiers and disabled men. The Minister of Labour pointed out the difficulties caused by some trade unions which refused to have anything to do with the training schemes. The number of disabled men able to compete with fit men was very small comparatively, and it was cruel to rob them of their chance. It is true this miserably selfish attitude is not a universal characteristic of the unions, and though the principle involved was defended by Labour Members, it is fairly certain it would not get substantial backing from workmen themselves, who are not lacking in sympathy any more than other sections of the community. There may be proper objections to overcrowding a trade with semi-skilled men, though even here this danger ought to be capable of adjustment. But why should a youth be prevented from continuing his training started before the war and compelled in some cases to start again at the beginning? Why should skilled ex-Service men who have acquired technical training in the Army be prevented from undergoing short training to equip themselves definitely for that work? And what justice is there in calling strikes because of the employment of fully competent ex-Service men, merely because they have not gone through a strictly trade-union routine? And are the youths who entered the Army with no skilled training to be debarred altogether from starting in the Ironmoulders' Union? Any difficulties which are likely to arise in economic conditions should be a matter of adjustment by the trade unions themselves, and it is hard to believe they will remain deaf to the moderately phrased appeals made to them in Parliament. It has always been considered an axiom that, so far as is possible, no man who fought should suffer the loss of opportunity through fighting, and that is not putting his real and solid claim upon the nation extravagantly high. It is entirely inconsistent to demand complete freedom of the people and at the same time to try to defend an autocracy of Labour—not, mind you, towards society in general, but towards Labour itself. There are many glaring instances in which trade unions have absolutely refused to consent to patriotic proposals of employers to train ex-soldiers to particular crafts on full trade-union rates of pay, even during training; this has been offered in several cases (for which the writer can vouch) in trades wherein there is a great scarcity of skilled labour. It is useless for Labour to gibe at employers when their own record will not bear examination. And what is the use of complaining of unemployment, when sections of labour are putting obstacles in the way of those very men who deserve well of their country? England, they aggressively agree, should be made a place "fit for heroes" to live in, but trade unions—no, that is not a fit place for heroes; that is different. Therefore, while the Ministry of Pensions does its best to train disabled men to earn their livings, some prominent unions refuse to admit them to membership when they are so trained. Trade unionism must make up its mind either to act up to its high professions, or to drop its professions and its attacks upon the materialistic selfishness of employers.

